# Camphor-Induced Seizures in Rats Increase the Potency of Gamma Oscillations During the Ictal Period, A Component that may Lead to Refractoriness in Seizure Control

**DOI:** 10.1007/s12640-026-00793-3

**Published:** 2026-03-24

**Authors:** Priscille Fidelis Pacheco Hartcopff, Clarissa Araujo da Paz, Luciana Eiró Quirino, Thaysa de Sousa Reis, Daniella Bastos de Araujo, Rafael dos Reis de Souza, Caio Gabriel da Silva Motta, Mateus Felipe Ferreira Araújo, Marcelo Victor dos Santos Brito, Murilo Farias dos Santos, Alisson Bruno Leite Lima, Axell Lins, Moisés Hamoy

**Affiliations:** Laboratory of Pharmacology and Toxicology of Natural Products- ICB- UFPA, Federal University of Pará, R. Augusto Corrêa, 01, Belém, Pará 66075-110 Brazil

**Keywords:** Camphor, Electrocorticography, Seizure model, Anticonvulsants, Wistar rats

## Abstract

Particularly in the evaluation of substances that can cause neuronal hyperexcitability, triggering refractoriness to antiepileptic drugs, this has always been of interest to science. This study investigated the neurobehavioral and electrocorticographic (ECoG) characteristics of seizures induced by camphorated oil in Wistar rats and compared them to those triggered by pentylenetetrazol (PTZ), a classical chemoconvulsant model. Male rats received intraperitoneal administration of camphor (470 mg/kg), and behavioral evolution was monitored to characterize seizure onset and progression. ECoG recordings from the motor cortex were acquired for 30 min to assess cortical oscillatory activity during ictal and interictal periods. Camphor administration elicited six distinct and rapidly evolving behavioral patterns culminating in generalized clonic seizures with loss of postural reflexes. ECoG analyses revealed cyclic high-power ictal discharges interspersed with low-power interictal activity, resembling PTZ-induced seizures but with lower potency. Evaluation of anticonvulsant efficacy demonstrated that camphor-induced seizures were refractory to phenobarbital and phenytoin, whereas diazepam and propofol significantly reduced β- and γ-band power and effectively controlled convulsive activity. These findings suggest that camphor produces a reproducible chemoconvulsant model characterized by prominent cortical excitability and partial pharmacoresistance, supporting its potential utility in neurotoxicological and neuropharmacological studies aimed at understanding seizure mechanisms and testing antiepileptic drug responsiveness.

**Supplementary Information:**

The online version contains supplementary material available at 10.1007/s12640-026-00793-3.

## Introduction

Epilepsy is a disorder of the central nervous system characterized by recurrent seizures, which are characterized by an imbalance between excitatory and inhibitory factors, different symptoms depending on the locus of seizure activity and multifactorial causes (Perks et al. 2012; Curia et al. 2014; Chang et al., 2017; Anwar et al. 2020; Falco-Walter 2020; Neligan et al. 2023). Among the known causes are electrolyte disorders, systemic diseases affecting the nervous system, hypersensitivity encephalopathy, organ failure, fever and poisoning (Beghi et al. 2010; Baluarte 2017; Falco-Walter 2020; Mewasingh et al. 2020; El-Dib et al. 2023; Van Bogaert 2022; Rivas-García et al. 2022).

Treatments for epilepsy include anticonvulsant drugs, resective surgery, and functional surgery, with medication being the main treatment. Antiepileptic drugs primarily target voltage-gated ion channels, such as the update, potassium, and calcium channels, to modulate neuron electrical discharge (Lin and Hsieh 2021). To achieve a better understanding of antiepileptic and epileptogenic drugs, some seizure models are used, such as pentylenetetrazole (PTZ), pilocarpine and kainate (Curia et al. 2008; Kebriaeezadeh et al. 2008; Klee et al. 2017; Samokhina and Samokhin 2018; Rusina et al. 2021., Landucci et al. 2022; Guo et al. 2022; Monteiro et al. 2024; Monteiro at al., 2024; Gawel et al. 2024). From this perspective, scientific research has revealed the existence of other substances with convulsant properties, including some essential oils (EOs), such as eucalyptus and camphor (Mathew et al. 2020).

*Cinnamomum camphora* (L.) J. Presl, a large tree belonging to the Lauraceae family, is native to southern China, Taiwan and Japan. This tree is planted to produce camphor (Kang et al. 2019; Boroujeni et al. 2022; Lee et al. 2022). It has been used for centuries around the world as a remedy to treat a variety of symptoms, such as anxiety, inflammation, infection, congestion, pain and irritation (Wang, 2018; Lee et al. 2022; Faizmiya et al. 2022). The study by Bahr et al. (2019) points out that camphor has a proconvulsant effect and Mathew et al. (2020) illustrates the similarity of its mechanism of action with that of PTZ. The use of camphor as a seizure-inducing agent was identified in the mid-18th century, as its use was fundamental at that time for the improvement of psychiatric clinical conditions, however, without proper knowledge of its pharmacological properties (Romero-Tapia and Gamboa-Bernal 2018).

Many studies with medicinal plants are conducted to investigate possible therapeutic effects, including the components of essential oils, which are natural, volatile and complex phytochemical mixtures that are extracted from aromatic plants as secondary metabolites (Xiao et al. 2021). Essential oils are a particular class of natural medicines obtained by distilling plant material to obtain a volatile and hydrophobic extract (Bahr et al. 2019).

On the other hand, the occurrence of episodes related to side effects unrelated to phytotherapy such as the appearance of convulsions in laboratory animals serve as a good indicator of a chemoconvulsant model as observed with camphor after parenteral application (Gibson, 1989; Ferreira et al. 2020; Mathew et al. 2020; Jabbour et al. 2023).

Thus, the objective of this study was to evaluate the potential of camphor as a chemoconvulsant model and to characterize the behavior of the animals during the evolution of the seizure, as well as to evaluate the refractoriness to treatment with anticonvulsant medications. This aims to create a new experimental tool in scientific research for a greater understanding of the underlying mechanisms that can induce epileptic seizures. The choice of Wistar rats is important because, in neurotoxicology and epileptogenesis studies, they offer a stable neurological response comparable to human models, which facilitates the extrapolation of findings and includes new models for the study of the underlying mechanisms revealed in seizures.

## Materials and Methods

### Animals

For the study, heterogeneous male Wistar rats (*n* = 108) aged between 9 and 10 weeks and weighing between 180 g and 210 g were used. They came from the Central Animal Facility of the Federal University of Pará ICB - UFPA and were allocated to the animal facility of the Laboratory of Pharmacology and Toxicology of Natural Products - LFTPN -ICB-UFPA.

The animals were acclimatized to laboratory conditions 5 days before the experimental manipulation. They were placed in boxes measuring 50 cm x 60 cm x 20 cm (height x width x depth) with wood shavings, filtered water and commercial feed with 32% protein from Purina. They were kept at an ambient temperature of 25–26 °C, in 12- hour light/12-hour dark cycles. All the experiments were carried out from 8:00 am to 11:00 am. The entire experimental protocol was by Animal Use Ethics Committee of the Federal University of Pará (CEUA/UFPA) No. 8,381,060,818 (ID 000975).

### Chemical Products

The drugs were purchased from the following sources: ketamine - König (Santana de Parnaíba, SP, Brazil); xylazine - Vallée (Montes Claros, MG, Brazil). Anticonvulsant drugs phenobarbital (Aventis-Pharma, Ribeirão Preto, SP, Brazil); phenytoin, diazepam (União Química, Embu-Guaçu, SP, Brazil), Injectable sodium valproate (Depacon) (Abbott – Laboratories of Brazil Ltda, São Paulo, Brazil), Propofol (Union Chemistry – Guarulhos- SP- Brazil), Local anesthetic Lidocaine hydrochloride 2% from the Cristália laboratory (Travessa Sinamomo 70, Bairro Arvoredo, Concórdia, SC, Brazil) Ketoprofen Injectable 100 mg Eurofarma Laboratory S.A (Rod. Pres. Castello Branco, 3565 - Itapevi - SP), the pro-convulsant drug Pentylenetetrazole (PTZ) (SIGMA) and camphorated oil company UCBVET 2.35 g/10 mL.

### Experimental Design

#### Experiment 1: Behavioral Assessment

The animals were evaluated according to the evolution of the seizures. The dose of camphor was calculated according to the time taken for the seizures to progress to generalized clonic seizures with loss of the posture reflex between 5 and 10 min. Thus, the dose used was 470 mg/kg via i.p. (0.2 ml/100 g of body weight). Thus, doses of 410 to 470 mg/kg i.p. were used, increasing by 20 mg at each dose until reaching the dose that induced seizures in less than 10 min in all animals tested. Therefore, the average latencies for the tested doses were 410 mg/kg (660.5 ± 113.3 s) (*n* = 9), 430 mg/kg (589.25 ± 123.8 s) (*n* = 9), 450 mg/kg (498.78 ± 133.6 s) (*n* = 9), and 470 mg/kg (384.9 ± 74.41 s) (*n* = 9).

Latency times were assessed for the following behaviors: (1) immobility; (2) head and neck tremor; (3) fanned tail; (4) forelimb clonus; (5) generalized clonic seizure without loss of posture reflex and (6) generalized clonic seizure with loss of posture reflex. Generalized seizure with loss of posture reflex. Observation lasted 10 min.

#### Experiment 2: Electrocorticographic Recording (EcoG) Surgery for Electrode Implantation

The animals were anaesthetized by intraperitoneal injection of a combination of 10% ketamine hydrochloride at a dose of 100 mg / kg i.p. and 2% xylazine hydrochloride at a dose of 5 mg / kg i.p. The depth of anesthesia was assessed using the pain reflex after the interdigital reflex test (pressure applied between the animal’s digits; if there are no somatic manifestations or changes in breathing, it indicates a surgical anesthetic plane).In cases where the reflex was still present, anesthetic supplementation was carried out with 1/4 of the dose initially applied. During anesthesia, the animals were positioned in a stereotactic apparatus for trichotomy of the head and application of the local anesthetic Lidocaine to the incised skin.

After asepsis with alcoholic iodopolivinylpyrolidone (PVPI) O, surgery was performed to expose the skull. Two bilateral holes were drilled into the rat’s skull with a dental drill. Activated silver electrodes (0.5 mm diameter tip exposure) were placed in the dura mater above the frontal cortex at breech coordinates − 0.96 mm and ± 1.0 mm lateral (Hamoy et al. 2018) in the motor cortex region. A reinforcement screw was fixed to the skull, and the electrodes were fixed with dental acrylic cement (self-curing acrylic).

After surgery, the animals were treated with ketoprofen 1 mg/kg subcutaneously for 24 h. The animals were kept in individual cages. On the fifth day after surgery, the animals underwent camphor treatment for the acquisition of ECoG recordings. All recordings were made inside the Faraday cage.

### Electrocorticographic Recording

Five days after the electrodes were implanted, the groups were administered the drugs according to the following design: (a) Control group (received the equivalent volume of 0.9% saline solution); (b) group treated with pentylenetetrazole 60 mg/kg i.p (diluted in 0.9% physiological saline solution.); (c) Group treated with camphorated oil 470 mg/kg i.p. The recordings lasted 30 min and nine animals were used for each experiment.

The recordings were evaluated in terms of their power in oscillations up to 40 Hz, including the following ranges of brain oscillations: Delta (1–4 Hz), Theta (4–8 Hz), Alpha (8–12 Hz), Beta (12–28 Hz) and Gamma (28–40 Hz).

### Experiment 3 - Performance of Anticonvulsants

To test the anticonvulsants, on the fifth day after electrode implantation, the groups received the following medications: phenobarbital (10 mg/kg i.p.) (*n* = 9), phenytoin (10 mg/kg i.p.)(*n* = 9), diazepam (10 mg/kg i.p.) (*n* = 9), sodium valproate (10 mg/kg i.p.) (*n* = 9), and propofol (10 mg/kg i.v.) (*n* = 9), administered intravenously into the lateral tail vein, 5 min before the application of camphor (479 mg/kg i.p.). For the administration of the anticonvulsants, the animals were placed inside a tube for physical restraint, to facilitate access for intravenous administration. This procedure was performed with a microneedle (BD).

To assess the control of seizures by anticonvulsants, the animals were treated with camphor followed by the administration of anticonvulsants; the recordings lasted 15 min. *N* = 9 was used for each group.

### Analysis of the Recordings

The recordings were obtained using a differential amplifier with high AC input impedance (Grass Technologies, Model P511) adjusted with 0.3 Hz and 3 kHz filtering, 2000x amplification, monitored with an oscilloscope (Protek, Model 6510) and continuously digitized at a rate of 1 kHz by a computer equipped with a data acquisition card (National Instruments, Austin, TX) ( Hamoy et al. 2018).

The seizures were characterized using the Python programming language (version 2.7) and the Signal ^®^ 3.0 program. For signal acquisition. Analyses were carried out at a frequency of up to 40 Hz and divided into bands according to Hamoy et al. (2018) into Beta (1–4 Hz), Theta (4–8 Hz), Alpha (8–12), Beta (12–28) and Gamma (28–40 Hz), to interpret the dynamics during seizure development.

### Euthanasia

After carrying out the experiments, the animals were euthanized with a lethal injection of 300 mg/kg i.p. of ketamine associated with 30 mg/kg i.p. of xylazine hydrochloride and 10 mg/kg i.p. of diazepam; this procedure was necessary to avoid suffering the animals.

### Statistical Analysis

To verify the homogeneity between the groups, Levine’s test was used through the Python program using the scipy.stats library. The results were submitted to descriptive statistics, such as mean and standard deviation. One-way analysis of variance (ANOVA) was used, followed by a Tukey test, followed by the standard deviation of the means. A significance index of *p < 0.05 was adopted. **p < 0.01, ***p < 0.001. The sample size was determined based on previous studies with other pro-convulsant substances such as cyaniol (Hamoy et al. 2018), which demonstrated consistent results using a sample of 9 participants in each group.

## Results

### Description of the Characteristic Behavior Related to Camphor-Induced Convulsion

**Fig. 1:**
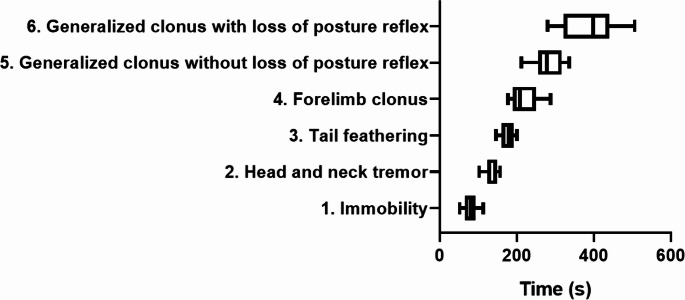
- Description of camphor-induced seizure-related behavior according to the latency for observing the behavior (*n* = 9)

After the administration of camphorated oil, the animals showed a rapid evolution of the seizure, displaying six behaviors. The change in behavior begins with immobility (akinesia) with an average latency of 78.89 ± 18.73 s, followed by head and neck tremors.

(135.9 ± 17.03 s) and tail stiffening (178 ± 17.59 s). The next behaviour involves the somatic system more intensely, revealing itself as clonias of the forelimbs (219.6 ± 37.37 s), the crisis evolving into a generalized clonic convulsion without loss of the posture reflex (281 ± 38.70 s) and with a worsening of the crises causing generalized clonic convulsions with loss of the posture reflex (384.9 ± 74.41 s). Each component of the behaviors recorded above is part of the subsequent behaviors (Fig. 1).

There are reports in the literature of *Cinnamomum camphora* inducing or provoking tonic-clonic seizures in patients with and without a history of seizures. Camphor induced generalized tonic-clonic seizures in 95% of the cases in a study with human patients with no previous history of seizures (*p* = 22) and caused generalized tonic- clonic seizures in 27.3% and localized or bilateral seizures in 15% of the other group of patients in the study, with a history of epilepsy or drug seizures (*p* = 33) (Mathew et al. 2020).

### Camphor-Induced Seizures have Similar Characteristics to the PTZ-Induced Chemoconvulsant Model

**Fig. 2:**
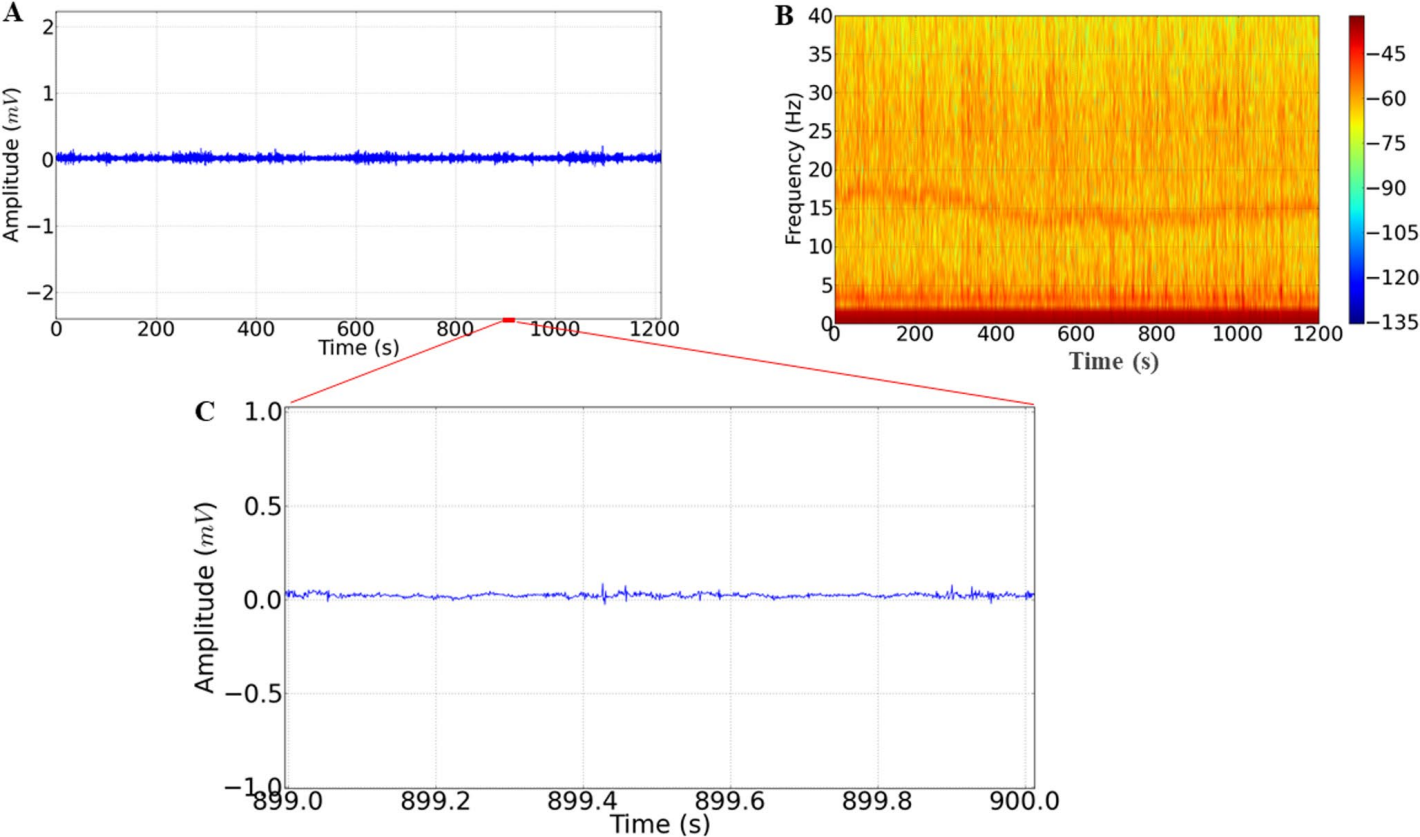
- ECoG recordings of the motor cortex of the control group (**A**), spectrogram of energy distribution up to 40 Hz (**B**), amplification of the recordings showing a duration of one second (899 to 900s) (**C**), recordings lasting 20 min

The readings of the ECoG recordings for the control group showed low amplitudes (0.05 mV), which keeps the recording regular, so the spectrogram shows higher levels of energy distributed at frequencies below 10 Hz (Fig. 2A and B). The amplification of the recording shows morphographic components related to the activity of the rat’s motor cortex, characterized by low amplitude with a predominance of low-frequency brain oscillations (Fig. 2C).

**Fig. 3:**
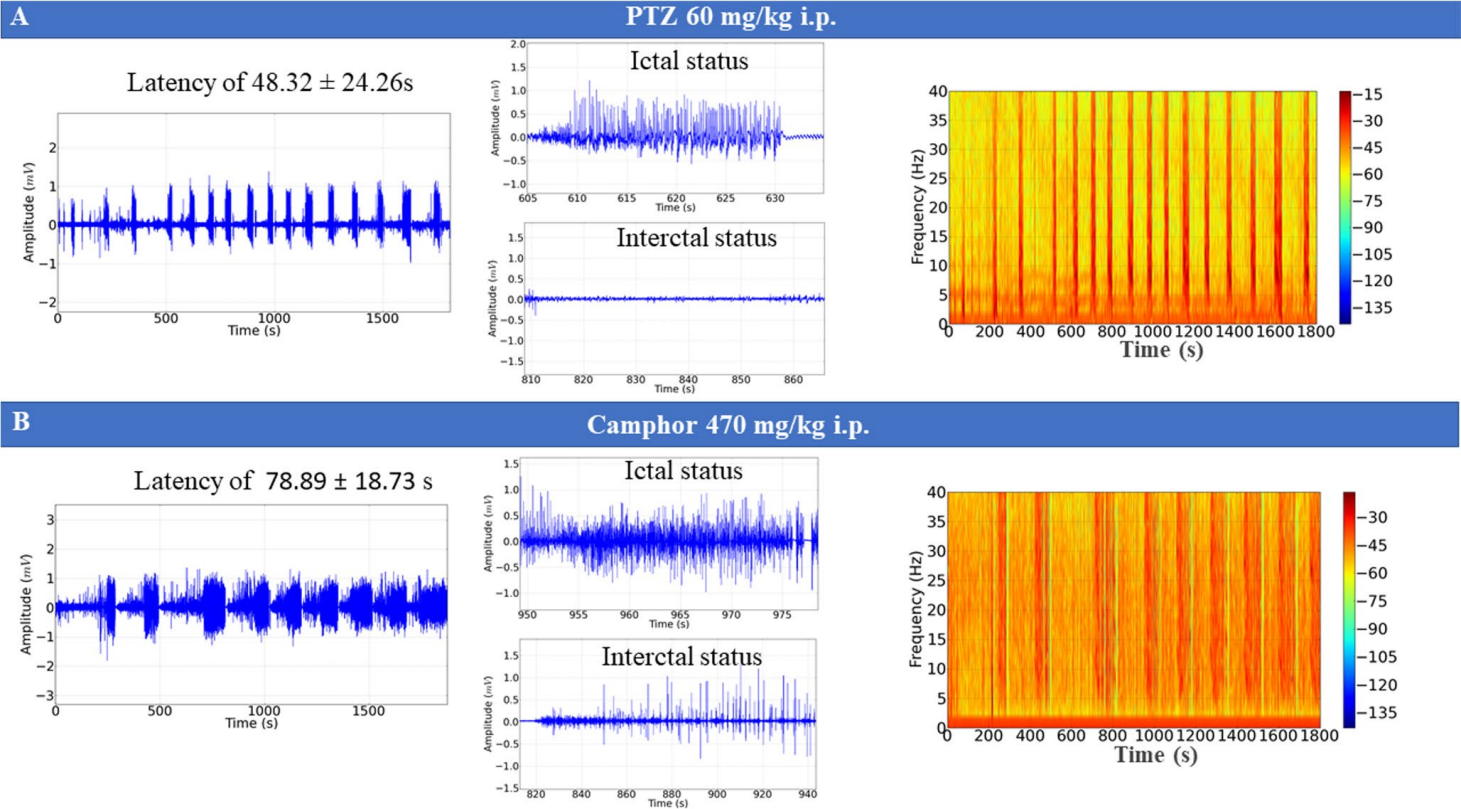
Demonstrations of ECoG recordings lasting 30 min. ECoG tracing of the group treated with pentylenetetrazole (PTZ) (left), amplification showing graphic components of ictal status (top center), amplification showing graphical elements of interictal status (bottom center) and spectrogram of energy distribution up to 40 Hz (right) (**A**). ECoG recording lasting 30 min of the camphorated oil-treated group (left), amplification of ictal status (top center), amplification of interictal status (bottom center) and power distribution spectrogram at frequencies up to 40 Hz (**B**)

The PTZ-treated group showed changes in the ECoG tracing with cyclic activity reaching amplitudes above 0.5 mV during ictal status, which are characteristic of seizures, and interictal status, which was characterized by a decrease in the amplitude of the recording to the range of 0.05 mV. After the application of PTZ, the latency for the onset of seizures was 48 ± 24s. The frequency spectrogram shows an increase in power in oscillations distributed up to 40 Hz (Fig. 3A).

The group treated with camphor oil showed intercalated ECoG tracings between ictal and interictal status, characterized by neuronal recruitment with an increase in field potential during ictal status, which showed an increase in the power of the recordings, and interictal status with a decrease in power; the latency period for occurrence was 78.89 ± 18.73 s. The spectrogram shows a greater distribution of energy during ictal status (Fig. 3B).

**Fig. 4:**
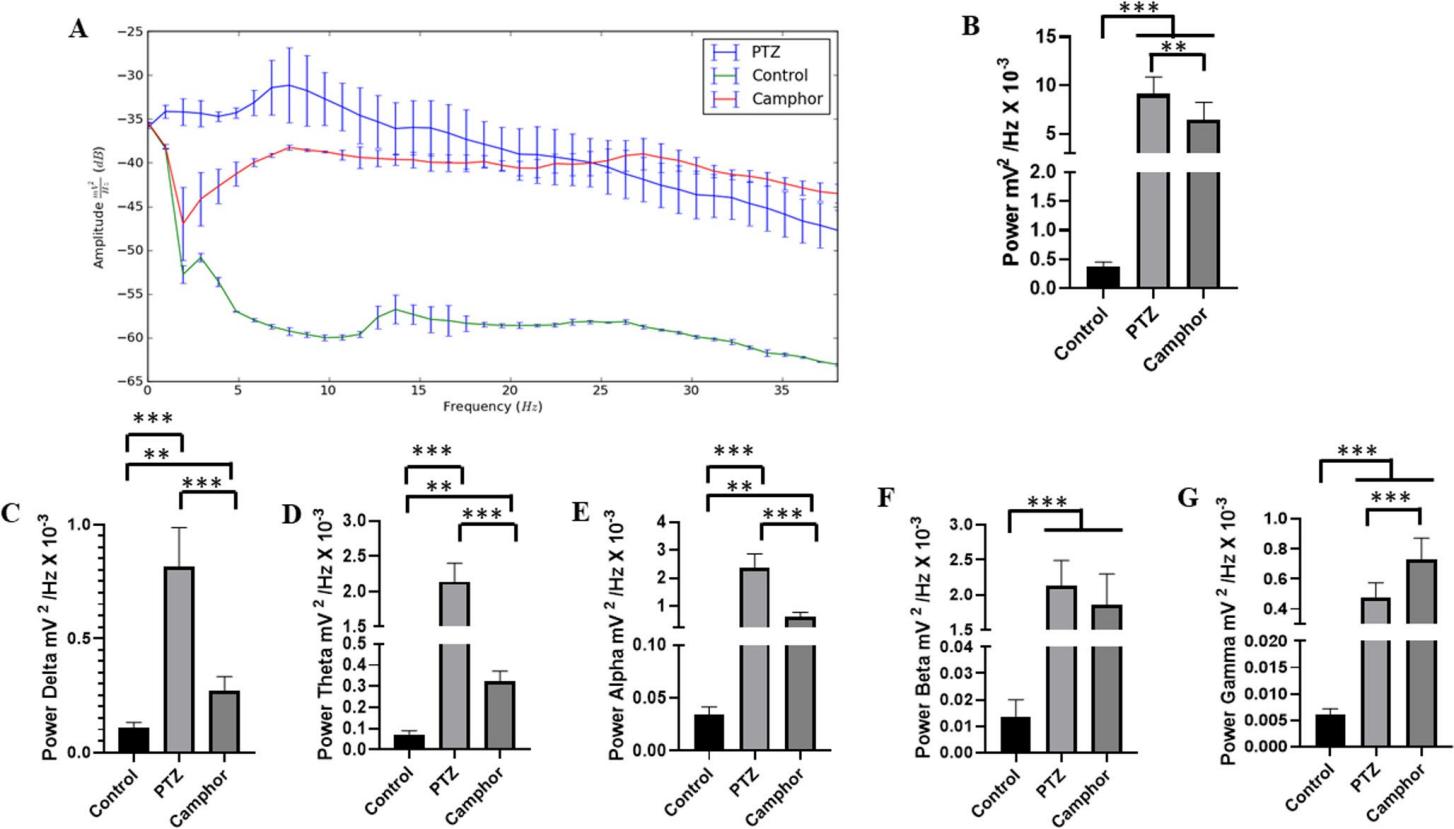
Spectral distribution of power at frequencies up to 40 Hz for the control, PTZ and camphor recordings (**A**); Graph representing the average linear power up to 40 Hz for the groups (**B**); Graphs of power distribution in brain oscillations up to 40 Hz: Delta ( 1–4 Hz) ( red line) (**C**), Theta (4–8 Hz) (black line) (**D**), Alpha (8–12 Hz) (yellow line) (**E**), Beta ( 12–28 Hz) (purple line) (**F**) and Gamma (28–40 Hz) (green line) (**G**). After ANOVA followed by Tukey, * *P* < 0.05 ** *P* < 0.01 ***P < 0.001, *n* = 9)

The spectral power distribution revealed greater amplitude in all brain waves in the camphor and PTZ groups compared to the control group (Fig. 4A). Power in the delta, theta and alpha frequencies was greater in the PTZ group, while gamma oscillations were more powerful in the camphor group. In this way, the average power in linear oscillations up to 40 Hz for the control group was 0.366 ± 0.086 mV² / Hz x 10^-^³ was lower than the groups treated with PTZ 9.11 ± 1.78 mV² / Hz x 10^-^³ and camphorated oil 6.46 ± 1.77 mV² / Hz x 10^-^³ (*p* = 0.001). There was a difference between the group treated with PTZ and camphor (*p* = 0.02) ( F_(2, 24)_ = 85.77; *p* < 0.0001) (Fig. 4B).

Mean analyses of the delta, theta, alpha, beta and gamma brain frequency bands showed an increase compared to the control group. Thus, PTZ and camphorated oil were able to induce disturbances in all brain waves, but the animals treated with camphorated oil showed greater power in gamma oscillations compared to the PTZ group (Fig. 4A).

For delta oscillations, the control group had a lower mean power (0.109 ± 0.022 mV² / Hz x 10-³) than the other groups PTZ (0.814 ± 0.171 mV² / Hz x 10-³) ( *p* = 0.001) and camphorated oil (0.271 ± 0.0620 mV² / Hz x 10-³) (*p* = 0.009). The group treated with PTZ had a higher mean than the group treated with camphorated oil ( *p* = 0.001) ( F_(2, 24)_ = 109.2; *p* < 0.0001) (Fig. 4C).

The mean power in theta oscillations for the control group (0.723 ± 0.017 mV² / Hz x 10-³) was lower than the PTZ (2.141 ± 0.263 mV² / Hz x 10-³) (*p* = 0.001) and camphorated oil (0.321 ± 0.048 mV² / Hz x 10^-^³) (*p* = 0.006) groups. The group treated with PTZ was superior to the group treated with camphorated oil (*p* = 0.001) ( F_(2, 24)_ = 478.5; *p* < 0.0001) (Fig. 4D).

In alpha oscillations, the control group had a mean of 0.033 ± 0.007 mV²/Hz x 10^-^.

³, which was lower than the groups treated with PTZ (2.35 ± 0.51 mV²/Hz x 10^− 3^) (*p* = 0.001) and camphorated oil (0.60 ± 0.153 mV²/Hz x 10^− 3^). The group treated with camphorated oil was inferior to the group treated with PTZ (*p* = 0.001) ( F_(2, 24)_ = 136; *p* < 0.0001) (Fig. 4E).

For beta oscillations, the control group had a mean of 0.013 ± 0.006 mV² / Hz x 10^-^³, which was lower than the treated groups (*p* = 0.001). The PTZ group had a mean power of 2.13 ± 0.354 mV²/Hz x 10^-^³ and was similar to the group treated with camphorated oil (*p* = 0.1928) ( F_(2, 24)_ = 112.1; *p* < 0.0001) (Fig. 4F).

For gamma oscillations, the control group (0.0059 ± 0.0011 mV²/Hz x 10^-^³) was lower than the other treated groups (*p* = 0.001). The camphor oil-treated group (0.730 ± 0.141 mV²/Hz x 10^-^³) was superior to the PTZ-treated group (0.471 ± 0.141 mV²/Hz x 10^-^³) (*p* = 0.001) ( F_(2, 24)_ = 118.7; *p* < 0.0001) (Fig. 4G).

**Fig. 5:**
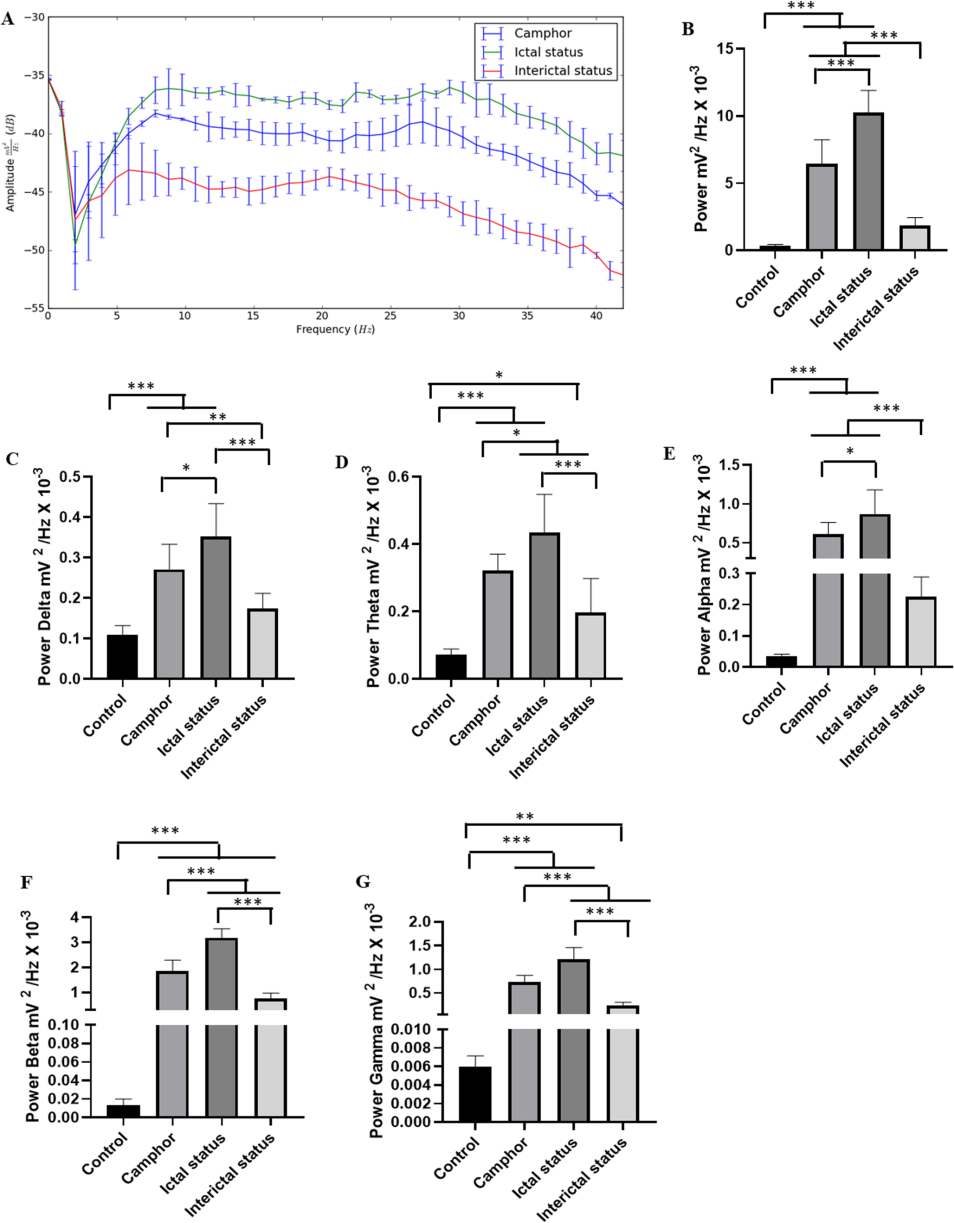
Spectral power distribution graph at frequencies up to 40 Hz in ECoG recordings during the seizure in the camphor oil-treated group and in the ictal status and interictal status periods (**A**); Linear power averages during ictal status and interictal status (**B**); Linear power average between groups with frequency in delta oscillations (1–4 Hz) (red line) (**C**); Average power distribution in theta frequency (4–8 Hz) (black line) (**D**); Graph of linear power distribution in the recordings between the groups in alpha oscillations (8–12 Hz) (yellow line) (**E**); Average power distribution in beta frequency (12–28 Hz) (purple line) (**F**); Graph of linear power distribution between the groups in gamma frequency (28–40 Hz) (green line) (**G**).(After ANOVA followed by Tukey, * *P* < 0.05 ** *P* < 0.01 ***P < 0.001, *n* = 9)

The spectral distribution graph of power at frequencies up to 40 Hz in the recordings of camphorated oil-induced seizures was divided into two distinct periods during the recording that occur in a cyclical manner, in the ictal status period a higher level of power was observed and in the interictal status period a lower level of power was observed in the oscillations (Fig. 5A). The average power for the control group (0.366 ± 0.086 mV²/Hz x 10^-^³) was similar to the interictal status group (*p* = 0.075). However, all groups had lower mean power during ictal status (10.25 ± 1.661 mV²/Hz x 10^-^³) ( F_(3, 32)_ = 116.4; *p* < 0.0001) (Fig. 5B).

For delta oscillations (1–4 Hz), the control group (0.109 ± 0.022 mV²/Hz x 10^-^³) had a mean power similar to the interictal period (*p* = 0.092), but was lower than the camphor oil (0.271 ± 0.062 mV²/Hz x 10^-^³) and ictal status (0.351 ± 0.081 mV²/Hz x 10^-^³) groups. The ictal status group was superior to all groups( F_(3, 32)_ = 32.94; *p* < 0.0001) (Fig. 5C).

The theta oscillations in the status ictal period (0.433 ± 0.114 mV²/Hz x 10^-^³) were higher than in the other groups. The interictal period had a higher mean (0.197 ± 0.100 mV²/Hz x 10^-^³) than the control group (0.072 ± 0.017 mV²/Hz x 10^-^³) ( F_(3, 32)_ = 33.76; *p* < 0.0001) (Fig. 5D).

For alpha oscillations, the mean power during ictal status (0.872 ± 0.308 mV²/Hz x 10^-^³) was higher than in the other groups. The average power for interictal status (0.225 ± 0.063 mV²/Hz x 10^-^³) was similar to the control group (*p* = 0.1129) ( F_(3, 32)_ = 41.72; *p* < 0.0001) (Fig. 5E).

The average power of beta oscillations during the ictal status period (3.18 ± 0.364 mV²/Hz x 10^-^³) was higher than the other groups. The control group (0.013 ± 0.0065 mV²/Hz x 10-³) was lower than the interictal status period (0.762 ± 0.212 mV²/Hz x 10^-^³)( F_(3, 32)_ = 184.5; *p* < 0.0001) (Fig. 5F).

For gamma oscillations, the control group (0.005 ± 0.001 mV²/Hz x 10^-^³) was lower than the other groups. The average power for the ictal status period (1.20 ± 0.25 mV²/Hz x 10^-^³) was higher than the other groups. The camphorated oil group (0.73 ± 0.141 mV²/Hz x 10^-^³) was superior to the interictal status group ( F_(3, 32)_ = 118.1; *p* < 0.0001) (Fig. 5G).

### Camphor had a Greater Preponderance in the Power of Beta and Gamma Oscillations and was Refractory to Treatment with Phenobarbital and Phenytoin

**Fig. 6:**
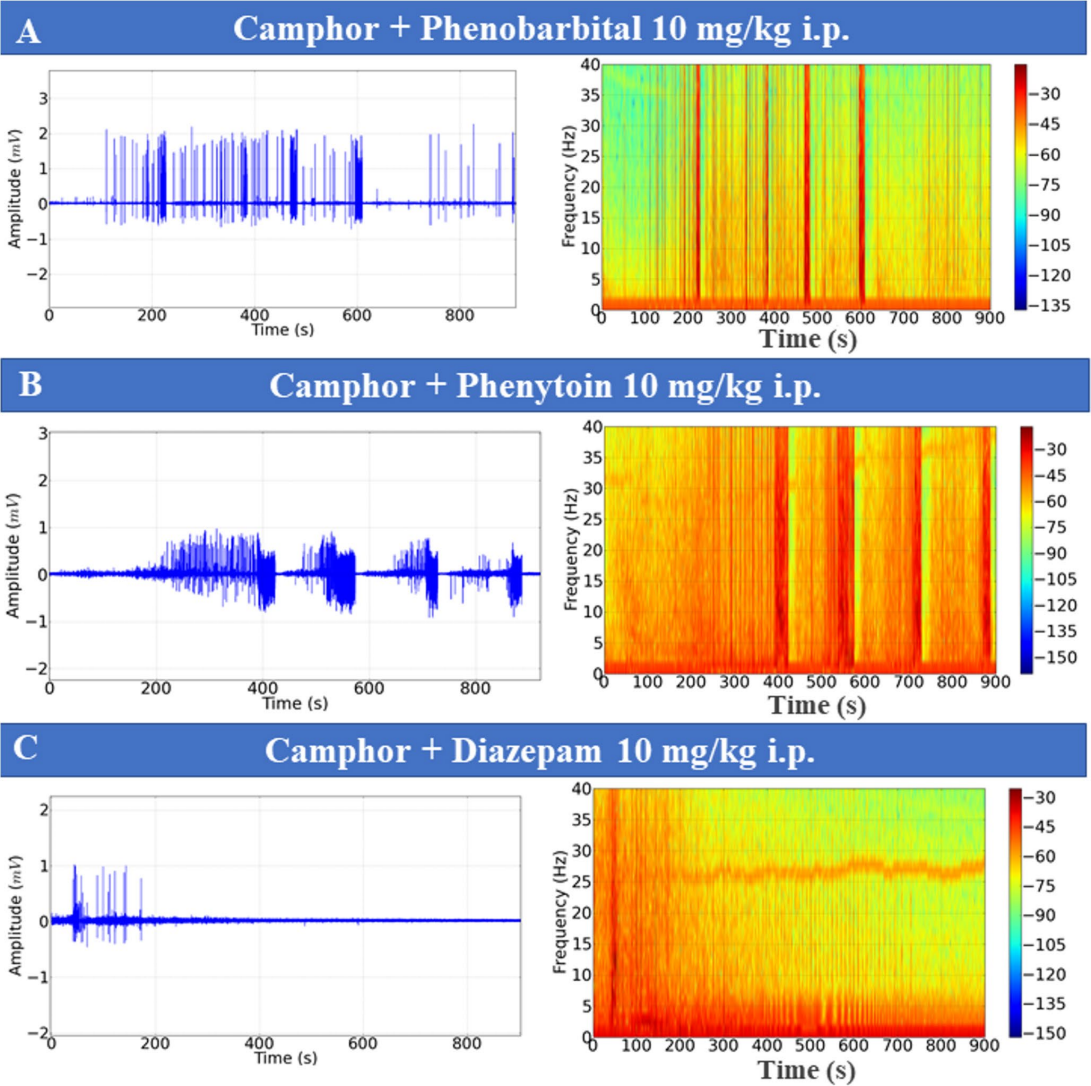

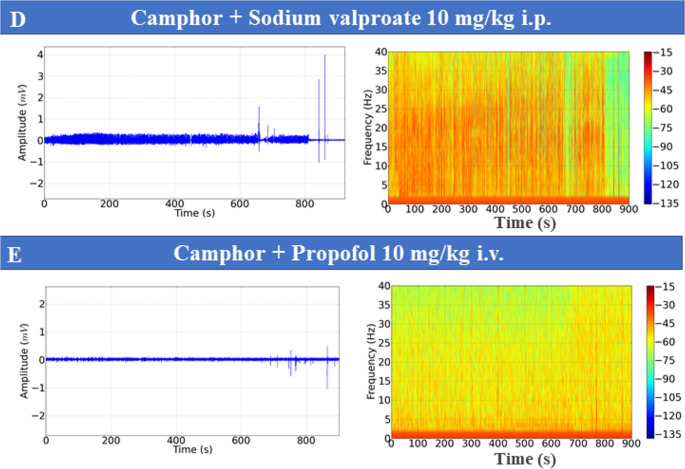

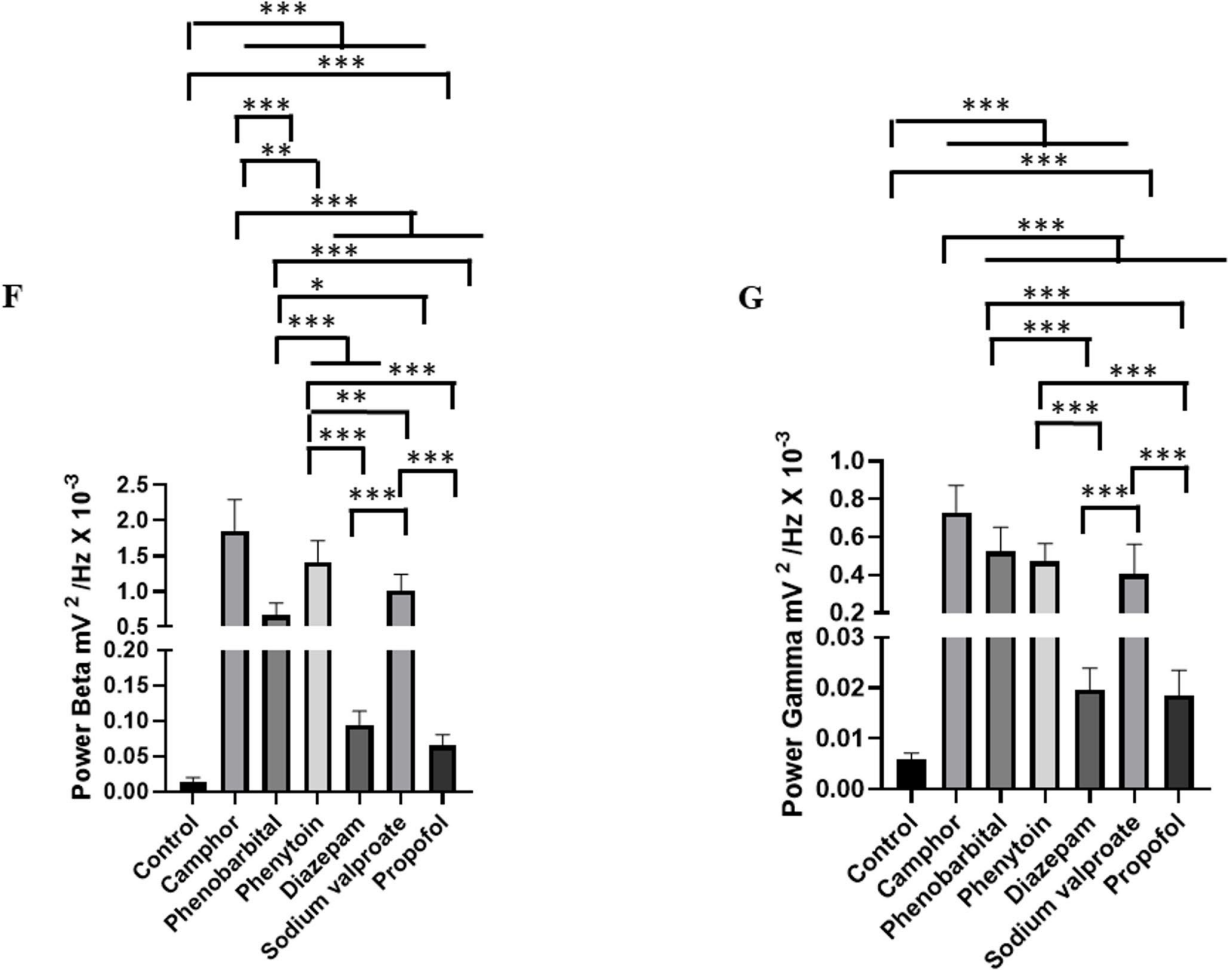
ECoG recordings demonstrating the action of anticonvulsants: Propofol (10 mg/kg, intravenous IV) (**A**), Diazepam (10 mg/kg IV) (**B**), Phenobarbital (10 mg/kg (IV) (**C**); Sodium valproate (10 mg/kg IV) (**D**), Phenytoin (10 mg/kg IV) (**E**), five minutes before the administration of camphor (470 mg/kg i.p.) and subsequently recorded for 15 min. Graph with mean power at beta (12–28 Hz) (**F**) and gamma (28–40 Hz) (**G**) frequencies. (After ANOVA followed by Tukey, * *P* < 0.05 ** *P* < 0.01 ***P < 0.001, *n* = 9)

To assess seizure control observed after camphorated oil treatment, the animals were treated with anticonvulsants, and then ECoGs were recorded, and their power in beta (12–28 Hz) and gamma (28–40 Hz) oscillations was assessed. Seizure control after phenobarbital use was observed 10 min after recording. During the first 10 min, periods of ictal and interictal states could still be identified (Fig. 6C).

Phenytoin and sodium valproate were ineffective in controlling seizures, with the ictal and interictal states maintained throughout the recording, demonstrating that the chemoconvulsant model induced by camphorated oil was refractory to the treatments (Fig. 6D and E). Seizure control was effectively achieved with diazepam and propofol, which indicated better protection for the animals (Fig. 6A and B), with propofol being more effective. Recording patterns obtained with the use of anticonvulsants are shown for beta and gamma oscillations (Figs. 6F and G).

The mean beta oscillation in the control group was 0.013 ± 0.006 mV²/Hz x 10^− 3^, similar to the group treated with Diazepam (*p* = 0.959) and propofol (*p* = 0.999), but was lower than the other groups. The group treated with phenytoin (1.41 ± 0.29 mV²/Hz x 10^− 3^) was superior to the other groups treated with anticonvulsants. The group treated with phenobarbital (0.659 ± 0.178 mV²/Hz x 10^− 3^) and lower than the camphorated oil group, treated with phenytoin and sodium valproate. The diazepam-treated group (0.0942 ± 0.0193 mV²/Hz x 10^− 3^ was similar to the propofol-treated group (*p* = 0.999) (F_(6, 56)_ = 90.02; *p* < 0.0001) (Fig. 6F). For gamma oscillations, the control group (0.005 ± 0.0011 mV²/Hz x 10^− 3^) was similar to the diazepam group (*p* = 0.998) and the propofol-treated group (*p* = 999). The phenobarbital group (0.523 ± 0.128 mV²/Hz x 10^− 3^) was similar to the phenytoin group (*p* = 0.923) and the sodium valproate-treated group (*p* = 0.169), but was inferior to the camphor group. The phenytoin-treated group (0.471 ± 0.094 mV²/Hz x 10^− 3^) was similar to the group treated with sodium valproate (*p* = 0.787). The group treated with Diazepam (0.0196 ± 0.004 mV²/Hz x 10^− 3^) was similar to the group treated with propofol (*p* = 0.999) (F_(6, 56)_ = 77.56; *p* < 0.0001) (Fig. 6G).

## Discussion

The behavioral data showed that camphor presented rapid evolution of convulsive behavior, six behaviors were identified culminating in generalized clonic convulsion with loss of the posture reflex (384.9 ± 74.41s) after application. The behaviours presented the following components: akinesia, head and neck tremor, tail stiffening, forelimb clonias, generalized clonic seizures without loss of posture reflex and generalized clonic seizures with loss of posture reflex, with the subsequent behaviours presenting components of the previous behaviours. These data indicate possible points of intersection between the chemoconvulsant model promoted by the administration of camphor and models traditionally studied with other chemical agents such as pentylenetetrazole and kainic acid, which represent suitable models for the study of refractory epilepsy to screen the activity of anticonvulsant drugs and which help to clarify the mechanisms involved in convulsive and epileptic processes (Lüttjohann et al. 2009; Van Erum et al., 2019; Ishikawa et al. 2020; de Almeida et al. 2020; Singh et al. 2021; Alharbi 2021; Dos Santos et al. 2021; Torun, Kilinc & Kilinc, 2022). Although some differences can be noted, such as the latency for the onset of seizure behavior and the dose, which shows that PTZ is more potent in triggering seizures when compared to camphor. A study by Hamoy et al. (2018) demonstrated the activity of the cequisterpene cunaniol obtained from wild Clibadium as a powerful GABA antagonist chemoconvulsant with greater potency than PTZ, indicating that natural substances can be used as new convulsive models. Although the seizure provoked by camphor has its own characteristics, such as the elevation of gamma oscillations, which may be linked to the refractory seizure pattern.

In the electrocorticogram, the morphographic elements showed a similarity to the recrudescence of activity that increases the field potential recorded, characterized as a salvo of potentials that results in the ictal period, which is characterized by high power in the recording, followed by a decrease in power corresponding to the post-ictal period; these morphographic characteristics can be observed in both camphor-induced and PTZ- induced seizures. This characteristic was also observed for cunaniol and caffeine-induced seizures (Hamoy et al. 2018; Eiró-Quirino et al. 2024).

In general, there is an increase in the power of brain oscillations up to 40 Hz during camphor- and PTZ-induced seizures when compared to the control group. The breakdown of brain oscillations indicates that Delta (1–4 Hz), Theta (4–8 Hz) and Alpha (8–12 Hz) powers were higher in the PTZ-treated group, but beta (12–28 Hz) oscillations were similar, and gamma power was higher for camphor, indicating a preponderance of forces in seizures induced at frequencies of (28–40 Hz). Leitão et al. (2022) studying seizures provoked by thiocolchicoside in rats observed an increase in the powers of cerebral oscillations in the motor cortex during the ictal period. These effects were also observed for seizures induced by nicotine and pilocarpine (Da Silva et al. 2022).

Regarding the characteristics of camphor-induced seizures, ictal and inter-ictal periods were observed. During the ictal period, the average power of the brain oscillations increased towards Beta and Gamma oscillations, with a characteristic higher frequency between (12–40 Hz). According to Naggar et al. (2020) field potential oscillations reflect repetitive firing of neuronal synaptic activity in certain areas of the brain that can be distinguished as slow (alpha, delta and theta), fast (beta and gamma). In this way, during the ictal period caused by camphor, fast powers predominate. These ictal characteristics have also been observed in the outbreak-suppression that occurs in children with West syndrome (Nariai et al. 2017). Which may be linked to refractoriness and include the camphor-induced seizure as a model for studies of refractory seizures. The use of camphor is contraindicated for susceptible patients.

When using anticonvulsants such as phenobarbital, phenytoin, diazepam, sodium valproate, and propofol to control epileptic seizures, and observing, after treatment with camphor, a reduction in the average power of beta and gamma oscillations, the best efficacy was observed with the application of propofol and diazepam, which positively modulate the response to GABA, hyperpolarizing the cell and hindering the generation of the action potential, reducing the average power of beta and gamma oscillations. Zhou et al. (2021) demonstrated, through a randomized clinical trial with epileptic seizure models using lithium and pilocarpine in adult rats, that diazepam, when administered within 10 min of the onset of the seizure, presents better results through the overall suppression of potency. Similarly, Rojas et al. (2018) demonstrated seizure interruption, but potency in the ~ 20–70 Hz bands (beta and gamma) returned hours later in rats with a seizure model using diisopropylfluorophosphate. Rossetti et al. (2024) stated that propofol can be used as an anticonvulsant in refractory seizures in severely ill patients, having a direct mechanism of action on the GABA receptor.

Phenobarbital was more effective in controlling mean beta potency compared to the phenytoin and valproic acid groups, this can be justified by the affinity with a specific site of GABAA receptors, causing a prolongation of the opening time of these channels, resulting in a greater influx of Cl^-^, as well as a reduction in the action of AMPA receptors and the blocking of L and N type Ca^2+^ channels. This corroborates other studies involving the control of seizures induced by pro-convulsant substances where treatment with diazepam was very effective (Mandhane et al. 2007; Akula et al. 2009; Mikutra-Cencora and Teitelbaum 2025). As was Propofol because it also binds to the allosteric site of the GABAA receptor, it has the particularity of a GABA-mimetic effect at higher concentrations, as well as the inhibition of NMDA receptors, breaking the cycle of hyperexcitability.

Although phenytoin delays the reactivation of Na^+^ channels in a use-dependent manner by inhibiting the most active neurons during seizures, in our study it did not show adequate efficacy in the ictal and interictal states, which may be explained by refractory blockades and alternative triggering pathways, such as Ca^+ 2^ channels and glutamate receptors. Furthermore, changes in pH and extracellular ions during the ictal state can alter the kinetics of Na^+^ channels, reducing the affinity of phenytoin, corroborating the study by West et al. (2022) who observed that phenytoin was not effective in suppressing resistance seizures, as well as in the interictal period.

As well as sodium valproate, which has a similar action in the context of blocking voltage-dependent Na^+^ channels, together with the stimulation of glutamate decarboxylase and blocking of T-type Ca^2+^ channels, In this context, Richardson et al. (2024) further highlighted that it is possible that it can increase the synaptic release of GABA, as well as modulate metabolic pathways, through the inhibition of alpha-ketoglutarate, increasing activity through the diversion of GABA.

In the present study, we describe the behavioral and electrophysiological characteristics during seizures provoked by the application of Camphor, using epidural ECoG recordings in the motor cortex of rats. Treatment with camphor resulted in changes in the graph-elements of the recording, which exhibited characteristics of convulsant activity, confirming that camphor is a potent convulsant agent in the rat brain. The induced seizures show a cyclical pattern with cellular recruitment triggering a salvo of potentials in the ictal and post-ictal periods, with a preponderance of beta and gamma oscillations during the ictal period. Control of camphor-induced seizures was better achieved with diazepam and propofol. In this way, the use of camphor makes it a suitable model for studies that address the mechanism involved in neurotransmission to assess the refractoriness of anticonvulsant drugs.

## Supplementary Information

Below is the link to the electronic supplementary material.

Supplementary Material 1

Supplementary Material 2

## Data Availability

The datasets generated are available from the corresponding author on reasonable request.
