# Supplementary material for: Camphor-Induced Seizures in Rats Increase the Potency of Gamma Oscillations During the Ictal Period, A Component that may Lead to Refractoriness in Seizure Control
Source: Neurotox Res. 2026 Mar 24;44(2):15. doi: 10.1007/s12640-026-00793-3 (PMC13013342; doi:10.1007/s12640-026-00793-3)
Supplement: Supplementary file 1 — Supplementary Material 1 [file 12640_2026_793_MOESM1_ESM.docx]

| 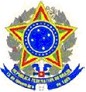 | **Ministry of Education - Brazil**  **Federal University of Pará**  Belém – PA- Brazil | 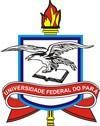 |
| --- | --- | --- |

**Manuscript title:** *Potential use of Camphor as a new chemoconvulsant model*

### **Highlights**

• **Camphor-induced seizures were systematically characterized** in Wistar rats through behavioral and electrocorticographic (ECoG) analyses, providing evidence of its neurotoxic convulsant potential.

• **ECoG recordings revealed high-power ictal discharges and increased β- and γ-band activity**, indicating cortical hyperexcitability comparable to pentylenetetrazol (PTZ) but with lower potency.

• **Camphor-induced seizures were partially refractory** to conventional anticonvulsants: phenobarbital and phenytoin were ineffective, whereas diazepam and propofol successfully suppressed β- and γ-power activity.

• **This model reproduces essential features of pharmacoresistant seizures**, supporting its application in neurotoxicology and neuropharmacology to investigate the mechanisms of neuronal excitability and drug responsiveness.

• **Camphor represents a novel, reproducible chemoconvulsant model** with translational value for the evaluation of antiepileptic drug efficacy and for studying the neurotoxic effects of essential oils and GABAergic system disruption.

**Sincerely,**

**Axell Lins, M.Sc.**
Corresponding Author
Laboratory of Pharmacology and Toxicology of Natural Products
Federal University of Pará (UFPA)
R. Augusto Corrêa, 01 – Guamá, Belém – Pará, Brazil
Tel: +55 91 98022-2421
Email: axell.ti20@gmail.com
