# Supplementary figures and images for: Camphor-Induced Seizures in Rats Increase the Potency of Gamma Oscillations During the Ictal Period, A Component that may Lead to Refractoriness in Seizure Control
Source: Neurotox Res. 2026 Mar 24;44(2):15. doi: 10.1007/s12640-026-00793-3 (PMC13013342; doi:10.1007/s12640-026-00793-3)

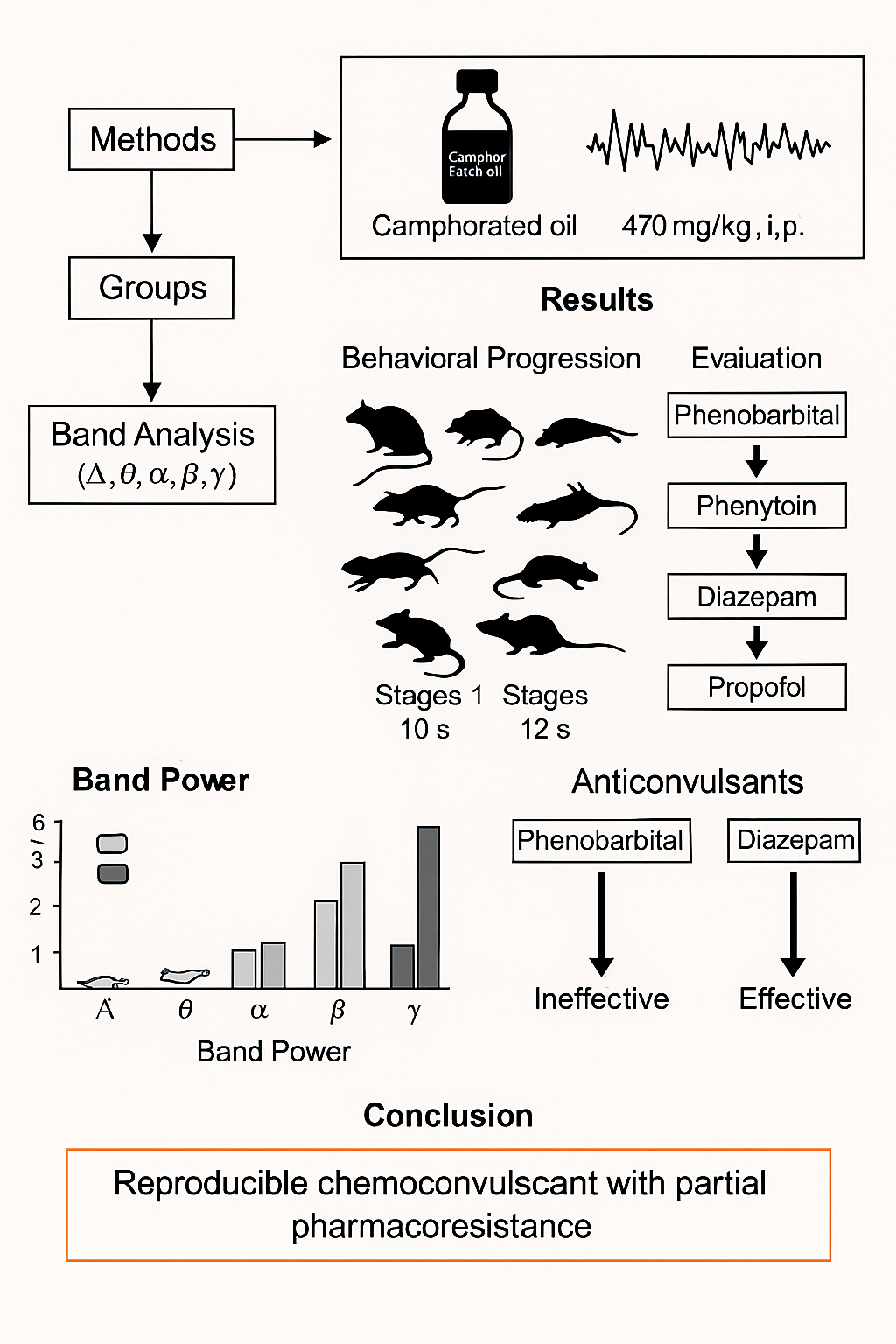

Supplement: Supplementary file 2 — Supplementary Material 2 [file 12640_2026_793_MOESM2_ESM.png]
